# Urinary biomarker panel for diagnosing patients with depression and anxiety disorders

**DOI:** 10.1038/s41398-018-0245-0

**Published:** 2018-09-19

**Authors:** Jian-jun Chen, Shun-Jie Bai, Wen-wen Li, Chan-juan Zhou, Peng Zheng, Liang Fang, Hai-yang Wang, Yi-yun Liu, Peng Xie

**Affiliations:** 1 Institute of Life Sciences, Chongqing Medical University, Chongqing, China; 2 Department of Neurology, The First Affiliated Hospital of Chongqing Medical University, Chongqing, China; 3 Institute of Neuroscience, Chongqing Medical University, Chongqing, China; 4 Joint International Research Laboratory of Reproduction & Development, Chongqing Medical University, Chongqing, China; 5 Department of Neurology, Yongchuan Hospital of Chongqing Medical University, Chongqing, China

## Abstract

Available data indicate that patients with depression and anxiety disorders are likely to be at greater risk for suicide. Therefore, it is important to correctly diagnose patients with depression and anxiety disorders. However, there are still no empirical laboratory methods to objectively diagnose these patients. In this study, the multiple metabolomics platforms were used to profile the urine samples from 32 healthy controls and 32 patients with depression and anxiety disorders for identifying differential metabolites and potential biomarkers. Then, 16 healthy controls and 16 patients with depression and anxiety disorders were used to independently validate the diagnostic performance of the identified biomarkers. Finally, a panel consisting of four biomarkers—*N*-methylnicotinamide, aminomalonic acid, azelaic acid and hippuric acid—was identified. This panel was capable of distinguishing patients with depression and anxiety disorders from healthy controls with an area under the receiver operating characteristic curve of 0.977 in the training set and 0.934 in the testing set. Meanwhile, we found that these identified differential metabolites were mainly involved in three metabolic pathways and five molecular and cellular functions. Our results could lay the groundwork for future developing a urine-based diagnostic method for patients with depression and anxiety disorders.

## Introduction

Depression and anxiety disorders are two major health concerns with similar core components while maintaining different meanings in various aspects^1^. Partly due to the overlap of symptoms in these two disorders, the presence of depression symptoms may serve to mask the presence of anxiety symptoms and vice versa. Previous studies reported that anxiety symptoms frequently coexist with depression symptoms^2,3^. Patients with depression and anxiety disorders have higher severity of illness, higher health care utilization, greater impairment in psychosocial functioning and lower quality of life than patients not suffering from comorbidity^4–6^. Moreover, available data indicate that patients with depression and anxiety disorders are likely to be at greater risk for suicide^7,8^. In addition, the presence of comorbidity could increase the chronicity of each disorder, slow the recovery and increase the likelihood of its recurrence^9,10^. These events often cause the patients to be treated for a longer time. Meanwhile, the unrecognized anxiety or depressive comorbidity has been shown to be related with an increased rate of psychiatric hospitalization^11^. Therefore, it is important to screen for comorbidity, which could help clinicians to make an effective treatment plan for patients. However, there are still no empirical laboratory methods to diagnose patients with depression and anxiety disorders. Currently, the diagnosis is made mainly according to the subjective identification of symptom clusters. However, this method could not be able to adequately characterize the heterogeneity, which unavoidably results in a considerable error rate^12,13^. An approach to deal with this limitation is to identify disease-specific biomarkers to support objective diagnostic laboratory tests.

Metabolomics has been widely used to identify the metabolic alterations in various disease states^14,15^. Currently, many analytical techniques have been developed for non-targeted metabolomic mappings, such as gas chromatography-mass spectroscopy (GC-MS) and nuclear magnetic resonance (NMR) spectroscopy. These analytical techniques have their own advocates and unique features. However, no single metabolomics platform could provide adequate coverage of the entire human metabolome in biosamples^16^. Previous studies have shown that the multiple metabolomics platforms could largely enhance the level of metabolome coverage^17,18^. Meanwhile, our previous study found that the combined application of NMR and GC-MS could yield a superior biomarker panel for bipolar disorder (BD) than applying each platform in isolation^19^.

Currently, many researchers have used metabolomics to successfully identify potential diagnostic biomarkers for neuropsychiatric disorders, such as BD, schizophrenia and autism^19–21^. Our group has identified several potential metabolite biomarkers for diagnosing major depressive disorder (MDD)^22,23^. However, few studies focus on the metabolic alterations in patients with depression and anxiety disorders. Therefore, in this study, we used the multiple metabolomics platforms (NMR and GC-MS) to study the metabolic changes in urine of patients with depression and anxiety disorders. Firstly, 32 patients and 32 age-, sex- and body mass index (BMI)-matched healthy controls (HCs) were used to identify the differential metabolites and potential biomarker panel. Secondly, 16 patients and 16 age-, sex- and BMI-matched HCs were further used to independently validate the diagnostic performance of the identified panel.

## Materials and methods

### Subject recruitment

The protocol of our study was reviewed and approved by the Ethical Committee of Chongqing Medical University. Patients were screened for depression and anxiety disorders in the baseline interview by two experienced psychiatrists using the DSM-IV (Diagnostic and Statistical Manual of Mental Disorders, 4th Edition)-based Composite International Diagnostic Interview (CIDI, version 2.1), which was a highly valid and reliable instrument for assessing depression and anxiety disorders^24^. The Hamilton Depression Rating Scale (HDRS) and Hamilton Anxiety Rating Scale (HAMA) were used to quantify the severity of depression and anxiety disorders, respectively^25,26^. The patients with HDRS scores of greater than 17 and HAMA scores of greater than 7 were recruited. Totally, 48 outpatients with depression and anxiety disorders were recruited from the psychiatric center of the First Affiliated Hospital of Chongqing Medical University. There were 40 patients with drug-naive first episode and 8 medicated patients with previous episodes. There were 39 patients with generalized anxiety disorder and 9 patients with panic disorder. During the interview, patients who met any one of the following criteria were excluded: (i) any pre-existing physical or other mental disorders (*n* = 87); (ii) pregnant or postpartum (within 1 year) women (*n* = 27); and (iii) illicit drug use (*n* = 31). During the same time period, the 48 age-, sex- and BMI-matched HCs were recruited from the Medical Examination Center. These HCs must have no DSM-IV Axis I/II disorders, previous lifetime history of neurological or psychiatric diseases or systemic medical illness. The number of patients seen by each experienced psychiatrist was almost the same. All participants provided the written informed consents before we collected the urine samples. The demographic and clinical details of the recruited subjects are described in Table 1.

**Table 1:** Demographic and clinical details of recruited subjects

Variables	Patients	HCs	*P* value^a^
Sample size	48	48	–
Medication (yes/no)	8/40	0/48	*p*<0.00001
Sex (male/female)	25/23	18/30	0.15
Age (year)^b^	31.83 (9.90)	31.96 (9.85)	0.95
BMI^b^	21.78 (2.45)	21.93 (2.53)	0.76
HDRS^b^	23.02 (4.25)	0.21 (0.65)	*p*<0.00001
HAMA^b^	16.71 (2.23)	0.71 (0.82)	*p*<0.00001

*HCs* healthy controls, *BMI* body mass index, *HDRS* Hamilton Depression Rating Scale, *HAMA* Hamilton Anxiety Rating Scale

^a^Two-tailed Student's test for continuous variables (age, BMI, HDRS and HAMA Scores); Chi-square analyses for categorical variables (medication and sex)

^b^Values expressed as the mean ± SD

### Two experimental sets

The included patients and HCs were divided into the training set and testing set in a 2:1 ratio. Finally, there were 32 patients and 32 HCs in the training set, and 16 patients and 16 HCs in the testing set. The training set was used to identify the differential metabolites and potential biomarker panel. It is critical to use the independent samples to further validate the diagnostic performance of the identified potential biomarker panel. Therefore, the testing set was used to independently validate its diagnostic performance. After overnight fasting, the morning midstream urine samples (9:00–10:00 am) of these subjects were collected and centrifuged at 1500 × *g* for 10 min. The obtained supernatant was divided equally and stored at −80 °C for later analysis. The procedures for NMR preparation and urine resonance assignments were performed according to our previous study^22^ and NMR databases^27^, respectively. The procedures for GC-MS preparation and GC-MS analysis were performed according to our previous studies^23,28^. The detailed information of GC-MS and NMR is described in the supplementary file 1.

### Statistical analysis

Firstly, in order to alleviate the effects of different samples, the original spectral data of metabolites were normalized to creatinine. Then, in order to eliminate the effects of different orders of magnitude, the data were further scaled to zero-mean and unit-variance. Finally, the obtained data were imported into SIMCAP+ 14.0 software for further analysis. The orthogonal partial least-squares discriminant analysis (OPLS-DA) was used to visualize the discrimination between HCs and patients with depression and anxiety disorders. The *R*^2^*X*, *R*^2^*Y* and *Q*^2^*Y* obtained from the default leave-one-out procedure were used to assess the quality of OPLS-DA model. The former two parameters and the last parameter were used to quantify the goodness of fit and assess the predictability of the model, respectively^29^. Meanwhile, a 300-iteration permutation test was conducted to find out whether there was non-randomness of separation between different groups. If the original *Q*^2^*Y* and *R*^2^*Y* values were higher than their corresponding values from the permutation test, then the built OPLS-DA model was considered to be valid.

In order to identify the differential metabolites responsible for samples separation, the coefficient loading plot of the built OPLS-DA model was analyzed^30^. Based on the number of samples in the training set, a correlation coefficient of |*r*| > 0.449 (equivalent to a *p* value < 0.01) was adopted as a cut-off value. Then, we used Pearson's correlation coefficient to assess the correlations between the identified differential metabolites, and used hierarchical clustering algorithm to show it. Meanwhile, to analyze the biological functions of these differential metabolites, we used the online software MetaboAnalyst 3.0 to conduct pathway analysis and Ingenuity Pathway Analysis (IPA) 9.0 to conduct core analysis. Also, we used the non-parametric Mann–Whitney *U*-test to detect whether or not these identified differential metabolites were still significantly changed between the two groups.

In addition, it should be more convenient and feasible to make a diagnosis in clinical practice by using a small number of metabolites. Therefore, these identified differential metabolites were further analyzed using a step-wise logistic-regression analysis based on the Akaike’s information criterion rule. The purpose of this analysis was to obtain a simplified metabolite biomarker panel^31^. To assess the diagnostic performance of this simplified panel, the receiver operating characteristic (ROC) curve analysis was conducted to quantify its ability in discriminating patients with depression and anxiety disorders from HCs in both training and testing sets.

## Results

### OPLS-DA model built

**Fig. 1:**
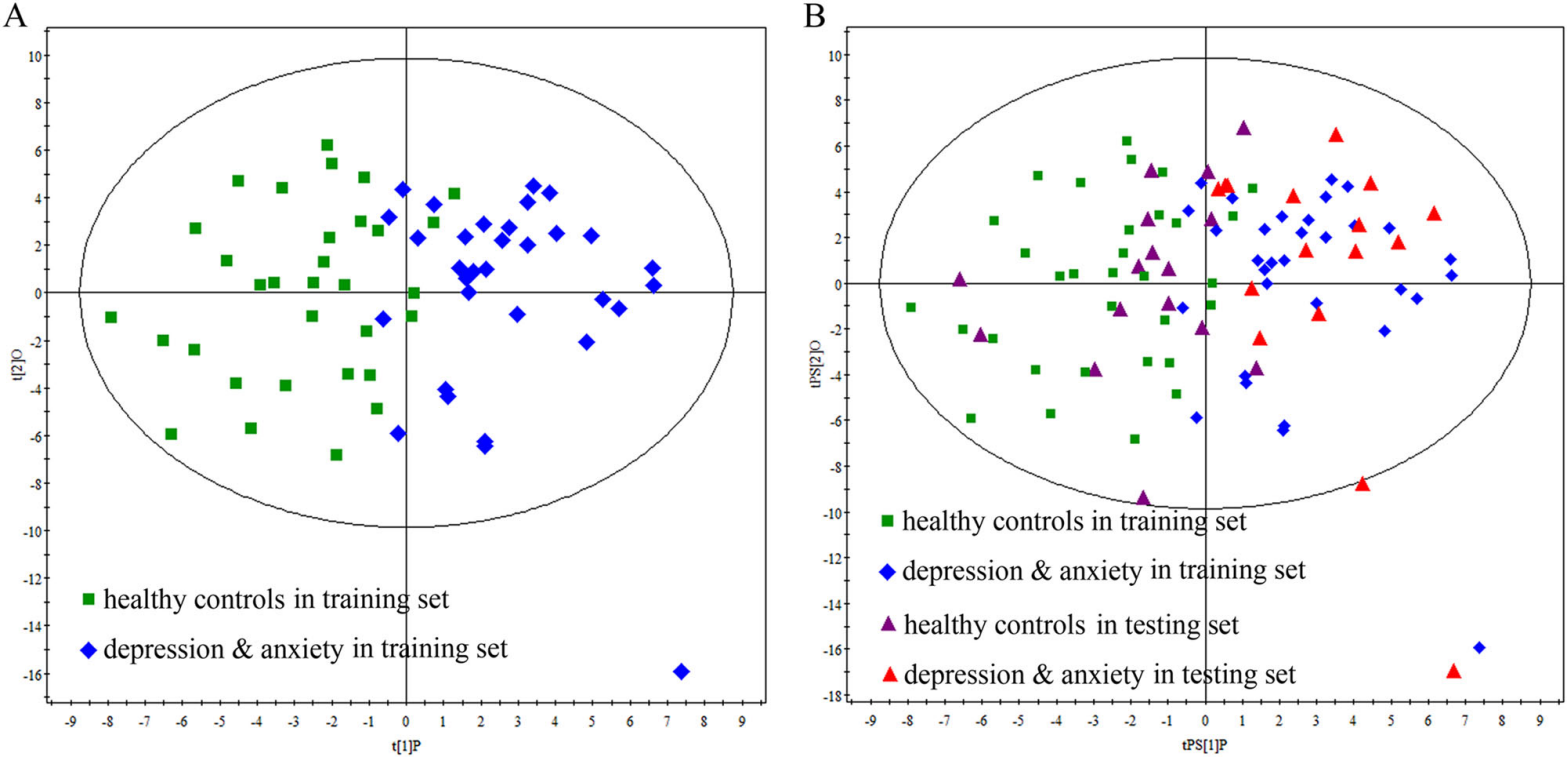
Metabolomic analysis of urine samples from the recruited subjects. **a** OPLS-DA model shows an obvious discrimination between patients with depression and anxiety disorders (blue diamond) and HCs (green box) in the training set. **b** T-predicted scatter plot shows that all patients with depression and anxiety disorders (red triangle) and 13 of the 16 HCs (purple triangle) in the testing set were correctly predicted

The samples in the training set were used to build the OPLS-DA model, which was used to explore the metabolic differences between HCs and patients with depression and anxiety disorders. The score plots of OPLS-DA model showed that the two groups were obviously separated with little overlap (*R*^2^*Y* = 61%, *Q*^2^*Y* = 45%; Fig. 1a). The positive values of these two parameters describing the model demonstrated a robust metabolic difference between the two groups. Furthermore, the permutation test also showed that the model was valid and not over-fitted as the original *Q*^2^ and *R*^2^ values were higher than their corresponding permutated values (for corresponding figure, see supplementary file 1). Meanwhile, the samples in the testing set were independently used to validate the diagnostic performance of this model. The T-predicted scatter plot showed that all patients with depression and anxiety disorders and 13 of the 16 HCs were correctly predicted by the model (Fig. 1b). These results indicated that this OPLS-DA model built by urinary metabolites held the promise to become an objective diagnostic tool for patients with depression and anxiety disorders.

### Differential metabolite identification

**Fig. 2:**
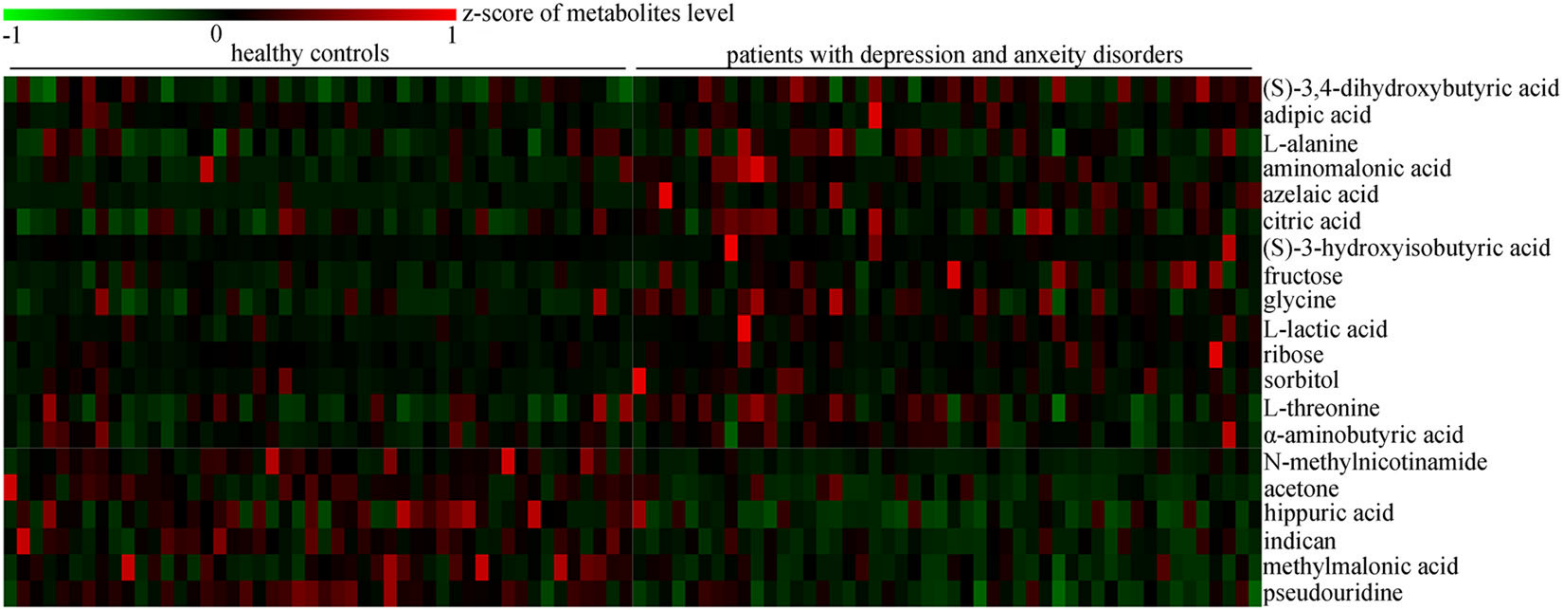
Heatmap of the identified differential metabolites. Green and red indicate the significantly lower and higher levels in patients with depression and anxiety disorders relative to HCs, respectively

**Fig. 3:**
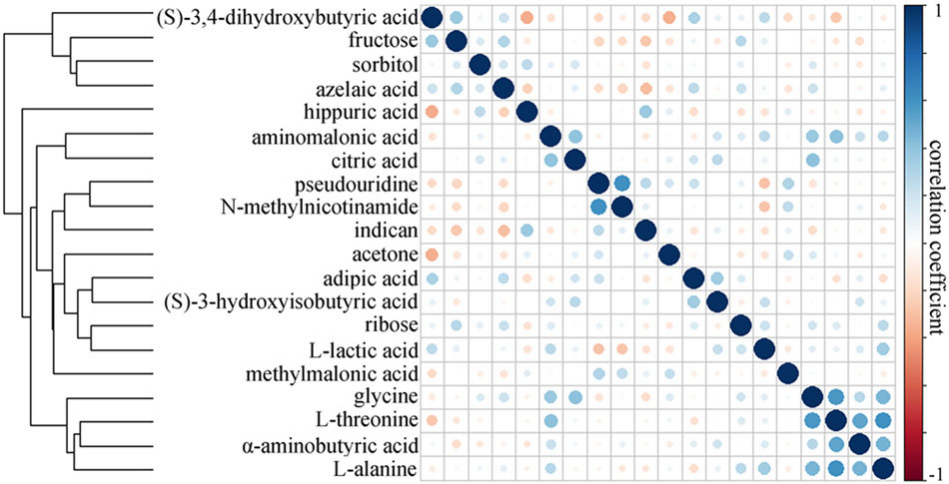
**Pearson's correlation coefficient and hierarchical clustering of the identified differential metabolites**

By analyzing the loading coefficient plots from the OPLS-DA model, we identified 20 differential metabolites (|*r*| > 0.449) responsible for the discrimination between HCs and patients with depression and anxiety disorders (Table 2). As compared to HCs, the patients with depression and anxiety disorders were characterized by higher levels of azelaic acid, aminomalonic acid, (*S*)-3-hydroxyisobutyric acid, fructose, sorbitol, l-lactic acid, glycine, l-alanine, citric acid, adipic acid, l-threonine, (*S*)-3,4-dihydroxybutyric acid, α-aminobutyric acid, and ribose, along with lower levels of acetone, methylmalonic acid, pseudouridine, indican, hippuric acid and *N*-methylnicotinamide (Fig. 2). Some of these metabolites have the relatively moderate correlations between each other (Fig. 3). The non-parametric Mann–Whitney *U*-test was then performed to validate the metabolic changes identified by the OPLS-DA model; the majority of these urinary metabolite levels remained significantly changed.

**Table 2:** Differential metabolites responsible for the discrimination of two groups

Metabolite	*P* value^a^	R^b^	FC^c^	Metabolic pathway
*N*-methylnicotinamide	5.32E−06	−0.693	−1.93337	Tryptophan–nicotinic acid metabolism
Acetone	0.008495	−0.501	−0.61032	Propanoate metabolism
(*S*)−3,4-dihydroxybutyric acid	0.002106	0.714	0.27378	Not found
(*S*)−3-hydroxyisobutyric acid	0.265085	0.523	0.841019	Valine, leucine and isoleucine degradation
Adipic acid	0.737113	0.46	0.347352	Degradation of aromatic compounds
l-alanine	0.096207	0.535	0.411909	Alanine metabolism
Aminomalonic acid	0.001461	0.77	1.070629	Not found
Azelaic acid	3.86E−05	0.662	1.841785	Lipid metabolism
Citric acid	0.088148	0.527	0.367736	Citrate cycle (TCA cycle)
Fructose	0.006761	0.525	0.66192	Starch and sucrose metabolism
Glycine	0.001052	0.654	0.561433	Glycine, serine and threonine metabolism
Hippuric acid	0.001068	−0.858	−1.02191	Tyrosine–phenylalanine pathway
Indican	5.62E−05	−0.8	−0.92295	Not found
l-lactic acid	0.001359	0.487	0.632744	Propanoate metabolism
Methylmalonic acid	0.007244	−0.671	−0.63621	Propanoate metabolism
Pseudouridine	9.33E−05	−0.477	−0.64546	Pyrimidine metabolism
Ribose	0.347268	0.612	0.214255	Pentose phosphate pathway
Sorbitol	0.113869	0.449	0.651624	Galactose metabolism
l-threonine	0.012509	0.505	0.330823	Glycine, serine and threonine metabolism
α-Aminobutyric acid	0.036643	0.486	0.217817	Cysteine and methionine metabolism

^a^*P* values were derived from non-parametric Mann–Whitney *U*-test

^b^Correlation coefficient was obtained from OPLS-DA with a threshold of 0.449, positive and negative values indicate higher and lower levels in patients with depression and anxiety disorders, respectively

^c^FC (fold change) positive and negative values indicate higher and lower levels in patients with depression and anxiety disorders, respectively

### Potential biomarker panel identification

The logistic-regression analysis was further used to obtain a simplified biomarker panel from these differential metabolites identified by the OPLS-DA model. The results showed that the most significant deviations between the two groups could be described by the following four urinary metabolites: *N*-methylnicotinamide, aminomalonic acid, azelaic acid and hippuric acid. The panel consisting of these four metabolites could yield an average accuracy of 92.2% in the training set and a predictive accuracy of 90.0% in the testing set. The discriminative model was: *P*(*Y* = 1) = 1/(1 + *e*−*y*); *y* = −114.277 × *N*-methylnicotinamide + 8.515 × aminomalonic acid + 7.106 × azelaic acid−0.075 × hippuric acid + 1.844. This model could calculate the probability of illness in each sample.

### Diagnostic performance assessment

**Fig. 4:**
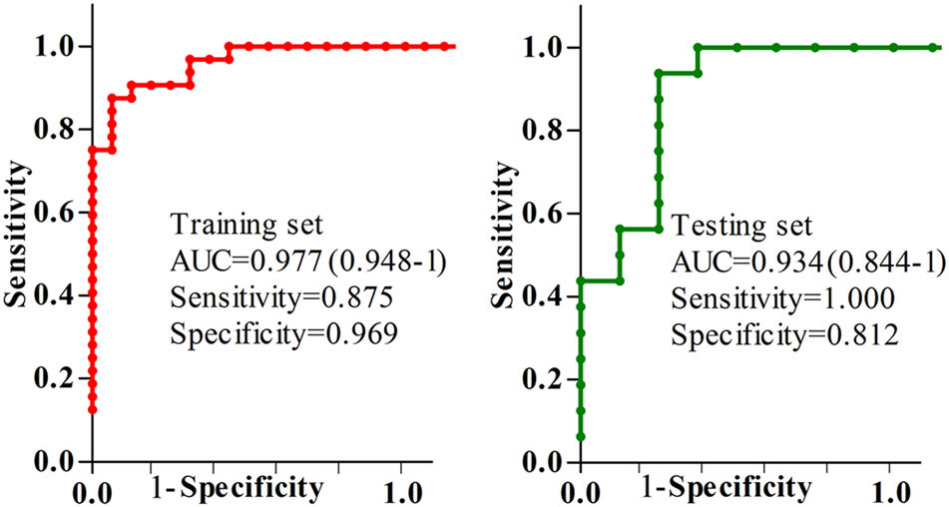
**Diagnostic performance of the simplified biomarker panel evaluated by ROC curve analysis**

The ROC curve analysis was further conducted to assess the diagnostic performance of this simplified panel (Fig. 4). In the training set, the results showed that the simplified panel was capable of distinguishing 32 patients with depression and anxiety disorders from 32 HCs with an area under the curve (AUC) of 0.977 (95% confidence interval (CI): 0.948–0.1). The sensitivity and specificity were 87.5% and 96.9%, respectively. Subsequently, this simplified panel was used to classify the blinded samples from the testing set. The ROC analysis yielded an AUC of 0.934 (95% CI: 0.844–0.1). The sensitivity and specificity were 100% and 81.2%, respectively. Meanwhile, these identified biomarkers had no sex specificity (for detailed information, see supplementary file 1). These results demonstrated the efficacy of this simplified panel in diagnosing patients with depression and anxiety disorders.

### Effects of medication on metabolites

There were eight medicated patients with depression and anxiety disorders. To determinate the homogeneity of metabolic phenotypes between the medicated and non-medicated patients, we first built the OPLS-DA model using the non-medicated patients and HCs (supplementary file 1). Then, the constructed OPLS-DA model was used to predict class membership of the medicated patients. The results showed that 7 of the 8 medicated patients were correctly predicted by the OPLS-DA model. These findings indicated that the metabolic phenotypes were significantly different between the non-medication patients and HCs, but not between the non-medicated and medication patients. Meanwhile, the panel of these four biomarkers could effectively discriminate medicated patients from HCs with an average accuracy of 96.4%. These results indicated that the medication might have little impact on metabolites in urine. Limited by the small sample size of medicated patients, this conclusion needs future studies to validate.

### Biological functions involved

**Fig. 5:**
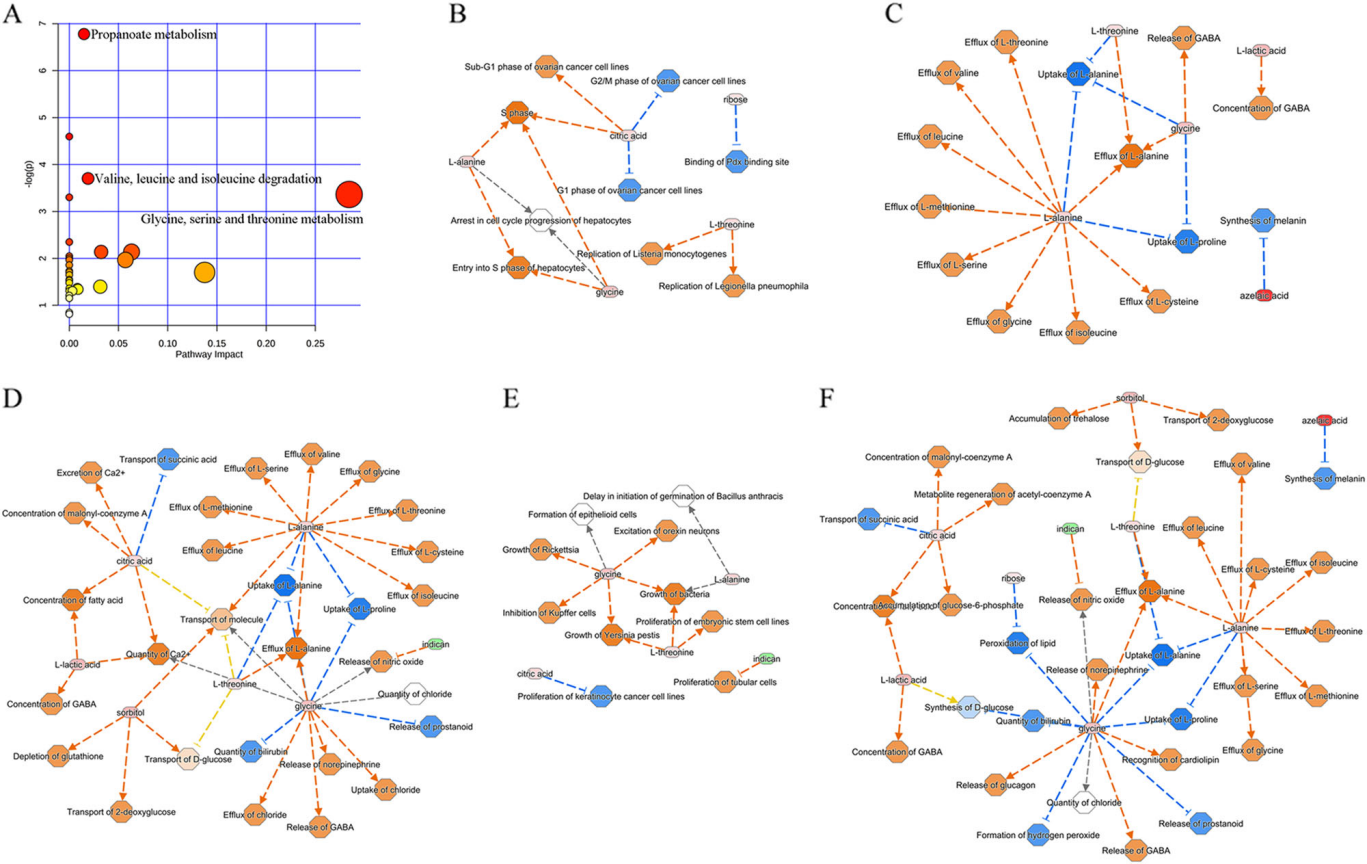
Pathways and biological functions of these differential metabolites mainly involved **a** The three affected metabolic pathways; **b** cell cycle; **c** amino acid metabolism; **d** molecular transport; **e** cellular growth and proliferation; and **f** small molecule biochemistry

The differential metabolites were imported into MetaboAnalyst 3.0 to conduct pathway analysis. We found that these 20 differential metabolites were mainly involved in three metabolic pathways (*p* value < 0.05, impact > 0): propanoate metabolism (*p* = 0.001, impact = 0.015) (methylmalonic acid, l-lactic acid, acetone); valine, leucine and isoleucine degradation (*p* = 0.024, impact = 0.019) (methylmalonic acid, (*S*)-3-hydroxyisobutyric acid); and glycine, serine and threonine metabolism (*p* = 0.034, impact = 0.284) (glycine, l-threonine) (Fig. 5a). Further analysis showed the possible increased activity of alanine-glyoxylate transaminase and 3-hydroxyisobutyrate dehydrogenase and decreased activity of aldehyde oxidase in patients with depression and anxiety disorders. Meanwhile, after importing these differential metabolites into IPA 9.0 to conduct core analysis, we found that these identified differential metabolites were significantly involved in the following top five molecular and cellular functions: cell cycle (Fig. 5b), amino acid metabolism (Fig. 5c), molecular transport (Fig. 5d), cellular growth and proliferation (Fig. 5e) and small molecule biochemistry (Fig. 5f).

## Discussion

In this study, the multiple metabolomics platforms were performed to explore the metabolic changes in patients with depression and anxiety disorders. Finally, the 20 differential metabolites were found, and a potential biomarker panel consisting of 4 urinary metabolite biomarkers (*N*-methylnicotinamide, aminomalonic acid, azelaic acid and hippuric acid) was identified. This panel could discriminate the patients with depression and anxiety disorders from HCs with AUCs of 0.977 and 0.934 in the training and testing set, respectively. These results demonstrated that this urinary biomarker panel might be a “good” classifier of HCs and patients with depression and anxiety disorders, and these differential metabolites could be helpful for future development of objective diagnostic methods.

Previous studies showed the consistently high comorbidity rates (ranged from 40% to 80%) for depression and anxiety disorders^32,33^. Comorbid disorders, especially comorbidity between depression and anxiety disorders, were more severe, carry more disability and had a greater persistence and duration than any single disorder alone^34^. This evidence indicated that the comorbidity might be a consistent predictor of chronicity. Due to the large impact of comorbidity on course and prognosis, the correct diagnosis of depression and anxiety disorders was very important as the first step in its prevention. If undiagnosed or unrecognized, the depression and anxiety disorders would contribute to high medical utilization in the primary care setting^35^. Dimatteo et al.^36^ reported that the unrecognized depression or anxiety disorder comorbidity could lead to a threefold increased likelihood of nonadherence to treatment. Some studies reported that patients with depression and anxiety disorders were less educated, had higher neuroticism scores and more often had a childhood trauma compared to patients with pure disorders^37,38^. However, the effects of these vulnerability and socio-demographic factors have not been often studied in concert with clinical characteristics. Here, we provide a potential novel method to objectively diagnose patients with depression and anxiety disorders.

Urine is a sterile and transparent fluid generated by kidneys. It contains high concentrations of urea, inorganic salts, ammonia, various water-soluble toxins, creatinine and urobilin. While the urine is largely viewed as a water product, it has a very high value as a diagnostic biofluid. In fact, urine is the first biofluid for clinically diagnosing alkaptonuria^39^. Nowadays, many researchers used metabolomics to study the urinary metabolites for identifying disease biomarkers. At present, up to 294 different human urinary metabolites could be possibly identified^17^. However, there is still no single platform that could quantify the entire human metabolome. Then, the identified disease biomarkers by single platform have unavoidable limitations. Therefore, to circumvent these limitations, more and more researchers used the combination of two or more platforms to help improve the metabolite coverage^40–42^. Here, the panel identified by the combined application of two platforms yielded very high AUC values in both training and testing sets, highlighting the diagnostic robustness of the identified biomarkers.

Notably, the significantly changed (*S*)−3-hydroxyisobutyric acid, adipic acid, l-alanine, citric acid, ribose and sorbitol levels were not found by the univariate analysis. However, the multivariate analysis still viewed them as differential metabolites responsible for the discrimination between HCs and patients with depression and anxiety disorders. Our previous study also found similar results, and even some non-significantly changed metabolites in univariate analysis were identified as potential biomarkers by the multivariate analysis^22^. This phenomenon could be explained by the highest predictive power of the discrimination model after the addition of these metabolites. These results fully showed the advantage of the multivariate analysis over the univariate analysis in finding the potential significance of subtle metabolic differences between different groups^43^.

Alanine-glyoxylate transaminase belonged to the family of transferases, and mainly participated in the glycine, serine and threonine metabolism and alanine and aspartate metabolism. Our study found the significantly increased levels of glycine, l-threonine and l-alanine, which might suggest the increased activity of this metabolic enzyme in patients. As the substrate of 3-hydroxyisobutyrate dehydrogenase, the significantly increased level of (*S*)-3-hydroxyisobutyric acid might indicate the increased activity of 3-hydroxyisobutyrate dehydrogenase. As the product of 3-hydroxyisobutyrate dehydrogenase, the level of methylmalonic acid semialdehyde might be increased due to the increased activity of 3-hydroxyisobutyrate dehydrogenase and its significantly increased substrate. Meanwhile, the methylmalonic acid semialdehyde was also the substrate of aldehyde oxidase. Considering the increased level of methylmalonic acid semialdehyde and significantly decreased level of methylmalonic acid (a product of aldehyde oxidase), there might exist decreased activity of aldehyde oxidase in patients with depression and anxiety disorders.

Zarate et al.^44^ reported that the glutamate propionic acid receptor in the propanoate metabolism pathway was a potential target for novel therapeutics for BD. Our previous study found that the propanoate metabolism pathway was significantly affected in BD patients^19^. Here, the results indicated that this pathway might also be significantly affected in patients with depression and anxiety disorders. Azelaic acid was a metabolite of phenylalanine long-chain fatty acids. Our previous study found that the gut microbiota had a role in lipid metabolism in the hippocampus of mice^45^, and the disturbed gut microbiota might have a causal role in the development of depression^46,47^. In this study, we found the significantly increased level of azelaic acid in patients with depression and anxiety disorders. Moreover, hippuric acid (also a metabolite of phenylalanine) and indican could be produced by bacterial metabolism in the intestinal tract. Then, these findings might highlight the potential involvement of gut microbiota variation in the development of depression and anxiety disorders. Meanwhile, we found that the synthesis of melanin, also a metabolite of phenylalanine, was inhibited by the significantly increased azelaic acid. A systematic review reported that the melatonin was effective for reducing preoperative anxiety symptoms and might also reduce postoperative anxiety symptoms in adults when given as premedication^48^. Therefore, the inhibition of the synthesis of melanin might be the self-protection mechanism of patients with depression and anxiety disorders.

*N*-methylnicotinamide was an end-product of nicotinamide metabolism, and the nicotinamide was involved in the tryptophan–nicotinic acid pathway^49^. Then, the significantly changed *N*-methylnicotinamide level observed here suggested a disturbance of tryptophan–nicotinic acid pathway activity in patients with depression and anxiety disorders. Meanwhile, the tryptophan was the biochemical precursor of both nicotinic acid and serotonin^21^. Therefore, the changed levels of downstream metabolites in nicotinic acid metabolism might indicate the disturbance of serotonin biosynthesis in patients with depression and anxiety disorders. This speculation could be supported by the previous findings that the altered serotonergic neurotransmission could contribute to the pathoetiology of depression and anxiety disorders^50,51^.

Aminomalonic acid has important biological implications, because its presence could moiety potentially impact calcium binding properties to protein^52^. Our previous study found that venlafaxine, an antidepressant of the selective serotonin-norepinephrine reuptake inhibitor class, could significantly decrease the level of aminomalonic acid in rat hippocampus^53^. Here, we found that its level was significantly increased in patients with depression and anxiety disorders. Meanwhile, the previous study found that the l-asparagine was a protective factor for mild depression^54^, and Milman et al.^55^ reported that aminomalonic acid was a strong inhibitor of l-asparagine synthetase. Therefore, these results might indicate that the aminomalonic acid might be a risk factor for depression and anxiety disorders.

Our two previous metabolomics studies were conducted to identify the potential biomarker panels for diagnosing MDD^22,23^. These two studies only focused on the MDD, and did not take anxiety disorder into consideration. The accuracies of NMR-based panel (5 metabolites)^22^ and GC-MS-based panel (6 metabolites)^23^ to diagnose the whole sample in this study were only 77.1% and 79.1%, respectively. These accuracies were much lower than that of the panel identified in this study to diagnose the whole sample (91.1%). Compared to the two previous panels, the panel in this study needed fewer metabolites but could yield a higher accuracy. Among the identified 20 differential metabolites, there were 8 metabolites (acetone, (*S*)-3,4-dihydroxybutyric acid, (*S*)-3-hydroxyisobutyric acid, adipic acid, aminomalonic acid, l-lactic acid, l-threonine and α-aminobutyric acid) that were firstly identified in patients with depression and anxiety disorders. These results indicated that there were divergent urinary metabolic phenotypes between patients with depression alone and patients with depression and anxiety disorders; then, a specific biomarker panel for patients with depression and anxiety disorders was still needed.

Among the four biomarkers identified here, the significantly decreased *N*-methylnicotinamide and hippuric acid levels, and increased azelaic acid level, were also found in patients with depression alone;^22,23^ the decreased *N*-methylnicotinamide level and increased azelaic acid level were also found in patients with BD alone^19^. Meanwhile, Lin et al.^56^ reported that the levels of *N*-methylnicotinamide and hippuric acid were also significantly changed in patients with postpartum depression. Hou et al.^57^ also found the significantly altered *N*-methylnicotinamide and hippuric acid levels in hepatitis B patients with depression. In addition, the level of aminomalonic acid was found to be significantly altered in a chronic unpredictable mild stress rat model of depression^58^. These results indicated that our four identified biomarkers were closely related with neuropsychiatric disorders. After validation and support by future large-scale studies, the panel of these four identified biomarkers might be used in clinical settings. Then, the clinicians could use this panel to correctly diagnose patients with depression and anxiety disorders which was helpful for reducing the financial burden on patients.

There are several limitations in the current study. Firstly, the number of recruited subjects was relatively small; however, the high values of AUC in both training and testing sets demonstrated that the identified biomarkers had good representativeness. Secondly, all patients with depression and anxiety disorders were from the same site and might have similar dietary habits, which might limit the general applicability of our conclusion. Thirdly, only the urine was used here, and future studies should further explore the other peripheral compartments to ensure the identified biomarkers being physiologically relevant to disease pathogenesis. Fourthly, although we found that the medicated and non-medicated patients had similar metabolic phenotypes, future studies with large samples were still needed to assess the effects of medication on urinary metabolites. Fifthly, we did not recruit patients with anxiety disorder who are lacking depressive symptoms, and thus the levels of these four biomarkers in these patients were unclear. Sixthly, we did not take the phases of menstrual cycle into account in female subjects which might have some effects on the metabolite abundance. Finally, the exclusion criteria for subjects did not include the history of neuropsychiatric disorders in their first-degree relatives. After excluding these subjects (two HCs and seven patients) to do re-analysis, we obtained the same biomarkers. However, future studies should take this point into consideration when identifying the potential biomarkers.

In conclusion, employing the multiple metabolomics platforms, a potential urinary biomarker panel for patients with depression and anxiety disorders was identified. This panel consisting of four metabolites—*N*-methylnicotinamide, aminomalonic acid, azelaic acid and hippuric acid—was capable of accurately distinguishing patients with depression and anxiety disorders from HCs in both training and testing sets. Our results could lay the groundwork for future developing a urine-based diagnostic method for patients with depression and anxiety disorders.

## Electronic supplementary material

Supplementary file 1

Original data

## References

[1] Zisberg A (2017). Anxiety and depression in older patients: the role of culture and acculturation. Int. J. Equity Health.

[2] Coplan JD, Aaronson CJ, Panthangi V, Kim Y (2015). Treating comorbid anxiety and depression: psychosocial and pharmacological approaches. World J. Psychiatry.

[3] Merikangas KR, Nakamura EF, Kessler RC (2009). Epidemiology of mental disorders in children and adolescents. Dialog. Clin. Neurosci..

[4] Olfson M (1997). Mental disorders and disability among patients in a primary care group practice. Am. J. Psychiatry.

[5] Brown C, Schulberg HC, Madonia MJ, Shear MK, Houck PR (1996). Treatment outcomes for primary care patients with major depression and lifetime anxiety disorders. Am. J. Psychiatry.

[6] Mclaughlin TP, Khandker RK, Kruzikas DT, Tummala R (2006). Overlap of anxiety and depression in a managed care population: prevalence and association with resource utilization. J. Clin. Psychiatry.

[7] Lepine JP, Chignon JM, Teherani M (1993). Suicide attempts in patients with panic disorder. Arch. Gen. Psychiatry.

[8] Kessler RC (2000). Lifetime panic-depression comorbidity in the National Comorbidity Survey. Br. J. Psychiatry J. Ment. Sci..

[9] Jacobson NC, Newman MG (2017). Anxiety and depression as bidirectional risk factors for one another: a meta-analysis of longitudinal studies. Psychol. Bull..

[10] Mathew AR, Pettit JW, Lewinsohn PM, Seeley JR, Roberts RE (2011). Co-morbidity between major depressive disorder and anxiety disorders: shared etiology or direct causation?. Psychol. Med..

[11] Sayers SL (2007). Psychiatric comorbidity and greater hospitalization risk, longer length of stay, and higher hospitalization costs in older adults with heart failure. J. Am. Geriatr. Soc..

[12] Mitchell AJ, Vaze A, Rao S (2009). Clinical diagnosis of depression in primary care: a meta-analysis. Lancet.

[13] Chen L, Eaton WW, Gallo JJ, Nestadt G (2000). Understanding the heterogeneity of depression through the triad of symptoms, course and risk factors: a longitudinal, population-based study. J. Affect Disord..

[14] Kaddurah-Daouk R, Kristal BS, Weinshilboum RM (2008). Metabolomics: a global biochemical approach to drug response and disease. Annu. Rev. Pharmacol. Toxicol..

[15] Zheng P (2016). Identification of sex-specific urinary biomarkers for major depressive disorder by combined application of NMR- and GC-MS-based metabonomics. Transl. Psychiatry.

[16] Williams R (2006). A multi-analytical platform approach to the metabonomic analysis of plasma from normal and Zucker (fa/fa) obese rats. Mol. Biosyst..

[17] Bouatra S (2013). The human urine metabolome. PLoS One.

[18] Li Y (2013). Metabonomics study of essential hypertension and its Chinese medicine subtypes by using gas chromatography-mass spectrometry and nuclear magnetic resonance spectroscopy. Evid. Based Complement. Alternat. Med..

[19] Chen JJ (2014). Combined application of NMR- and GC-MS-based metabonomics yields a superior urinary biomarker panel for bipolar disorder. Sci. Rep..

[20] Yang J (2013). Potential metabolite markers of schizophrenia. Mol. Psychiatry.

[21] Yap IK (2010). Urinary metabolic phenotyping differentiates children with autism from their unaffected siblings and age-matched controls. J. Proteome Res..

[22] Zheng P (2013). Identification and validation of urinary metabolite biomarkers for major depressive disorder. Mol. Cell. Proteomics.

[23] Zheng P (2013). A novel urinary metabolite signature for diagnosing major depressive disorder. J. Proteome Res..

[24] Kessler RC (2013). Composite International Diagnostic Interview screening scales for DSM-IV anxiety and mood disorders. Psychol. Med..

[25] Potts MK, Daniels M, Burnam MA, Wells KB (1990). A structured interview version of the Hamilton Depression Rating Scale: evidence of reliability and versatility of administration. J. Psychiatr. Res..

[26] Hamilton M (1959). The assessment of anxiety states by rating. Br. J. Med. Psychol..

[27] Beckwith-Hall BM (1998). Nuclear magnetic resonance spectroscopic and principal components analysis investigations into biochemical effects of three model hepatotoxins. Chem. Res. Toxicol..

[28] FS ShaoWH (2013). Metabolomic identification of molecular changes associated with stress resilience in the chronic mild stress rat model of depression. Metabolomics.

[29] Mahadevan S, Shah SL, Marrie TJ, Slupsky CM (2008). Analysis of metabolomic data using support vector machines. Anal. Chem..

[30] Cloarec O (2005). Evaluation of the orthogonal projection on latent structure model limitations caused by chemical shift variability and improved visualization of biomarker changes in 1H NMR spectroscopic metabonomic studies. Anal. Chem..

[31] Oikonomopoulou K (2008). Prediction of ovarian cancer prognosis and response to chemotherapy by a serum-based multiparametric biomarker panel. Br. J. Cancer.

[32] Graaf RD, Bijl RV, Spijker J, Beekman ATF, Vollebergh WAM (2003). Temporal sequencing of lifetime mood disorders in relation to comorbid anxiety and substance use disorders. Social. Psychiatry Psychiatr. Epidemiol..

[33] Jacobi F (2004). Prevalence, co-morbidity and correlates of mental disorders in the general population: results from the German Health Interview and Examination Survey (GHS). Psychol. Med..

[34] Lamers F (2011). Comorbidity patterns of anxiety and depressive disorders in a large cohort study: the Netherlands Study of Depression and Anxiety (NESDA). J. Clin. Psychiatry.

[35] Hirschfeld RM (2001). The comorbidity of major depression and anxiety disorders: recognition and management in primary care. Prim. Care Companion J. Clin. Psychiatry.

[36] Dimatteo MR, Lepper HS, Croghan TW (2000). Depression is a risk factor for noncompliance with medical treatment: meta-analysis of the effects of anxiety and depression on patient adherence. Arch. Intern. Med..

[37] Hovens JG (2010). Childhood life events and childhood trauma in adult patients with depressive, anxiety and comorbid disorders vs. controls. Acta Psychiatr. Scand..

[38] Weinstock LM, Whisman MA (2006). Neuroticism as a common feature of the depressive and anxiety disorders: a test of the revised integrative hierarchical model in a national sample. J. Abnorm. Psychol..

[39] Garrod AE (1996). The incidence of alkaptonuria: a study in chemical individuality. 1902. Mol. Med..

[40] Law WS (2008). Metabonomics investigation of human urine after ingestion of green tea with gas chromatography/mass spectrometry, liquid chromatography/mass spectrometry and (1)H NMR spectroscopy. Rapid Commun. Mass Spectrom..

[41] Chen JJ (2017). Differential urinary metabolites related with the severity of major depressive disorder. Behav. Brain Res..

[42] Chen JJ (2015). Divergent urinary metabolic phenotypes between major depressive disorder and bipolar disorder identified by a combined GC-MS and NMR spectroscopic metabonomic approach. J. Proteome Res..

[43] MacIntyre DA (2010). Serum metabolome analysis by 1H-NMR reveals differences between chronic lymphocytic leukaemia molecular subgroups. Leukemia.

[44] Zarate CA, Singh J, Manji HK (2006). Cellular plasticity cascades: targets for the development of novel therapeutics for bipolar disorder. Biol. Psychiatry.

[45] Chen JJ (2017). Effects of gut microbiota on the microRNA and mRNA expression in the hippocampus of mice. Behav. Brain Res..

[46] Zheng P (2016). Gut microbiome remodeling induces depressive-like behaviors through a pathway mediated by the host’s metabolism. Mol. Psychiatry.

[47] Chen J (2018). Sex differences in gut microbiota in patients with major depressive disorder. Neuropsychiatr. Dis. Treat..

[48] Hansen MV, Halladin NL, Rosenberg J, Gogenur I, Moller AM (2015). Melatonin for pre- and postoperative anxiety in adults. Cochrane Database Syst. Rev.

[49] Lester G (1971). End-product regulation of the tryptophan-nicotinic acid pathway in Neurospora crassa. J. Bacteriol..

[50] Belmaker RH, Agam G (2008). Major depressive disorder. N. Engl. J. Med..

[51] Senkowski D, Linden M, Zubragel D, Bar T, Gallinat J (2003). Evidence for disturbed cortical signal processing and altered serotonergic neurotransmission in generalized anxiety disorder. Biol. Psychiatry.

[52] Van Buskirk JJ, Kirsch WM, Kleyer DL, Barkley RM, Koch TH (1984). Aminomalonic acid: identification in Escherichia coli and atherosclerotic plaque. Proc. Natl. Acad. Sci. USA.

[53] Shunjie Bai QH (2017). Brain region-specific metabolite networks regulate antidepressant effects of venlafaxine. RSC Adv..

[54] Mayoral-Mariles A (2012). Plasma amino acid levels discriminate between control subjects and mildly depressed elderly women. Arch. Med. Res..

[55] Milman HA, Muth R, Cooney DA (1979). Aminomalonic acid and its congeners as potential in vivo inhibitors of L-asparagine synthetase. Enzyme.

[56] Lin L, Chen X, Liu R (2017). Novel urinary metabolite signature for diagnosing postpartum depression. Neuropsychiatr. Dis. Treat..

[57] Hou LJ (2015). Urinary metabonomics for diagnosis of depression in hepatitis B virus-infected patients. Iran. Red. Crescent Med. J..

[58] Li J (2014). Peripheral blood mononuclear cell-based metabolomic profiling of a chronic unpredictable mild stress rat model of depression. Mol. Biosyst..

