# Supplementary material for: Urinary biomarker panel for diagnosing patients with depression and anxiety disorders
Source: Transl Psychiatry. 2018 Sep 19;8:192. doi: 10.1038/s41398-018-0245-0 (PMC6145889; doi:10.1038/s41398-018-0245-0)
Supplement: Supplementary file 1 [file 41398_2018_245_MOESM1_ESM.docx]

**Urinary biomarker panel for diagnosing patients with depression and anxiety disorders**

Chen Jian-jun, Bai Shun-Jie, Li Wen-wen, Zhou Chan-juan, Zheng Peng, Fang Liang, Wang Hai-yang, Liu Yi-yun, Xie Peng

**Permutation test**

We used the 300-iteration permutation test to find out whether there was non-randomness of separation between different groups. If the original Q^2^Y and R^2^Y values were higher than their corresponding values from the test, then the built OPLS-DA model was considered valid. As shown in figure 1, the permutation test showed that the model was valid and not over-fitted as the original Q^2^ and R^2^ values were higher than their corresponding permutated values.


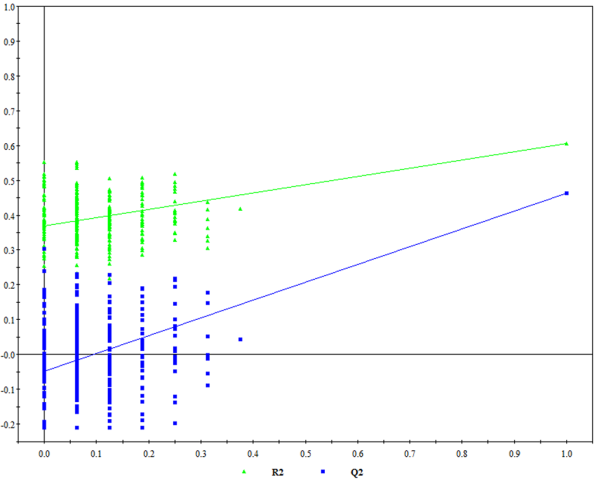


**Figure 1 corresponding figure from 300-iteration permutation test**

**Sex specificity**

In our previous studies, we successfully identified the sex-specific urinary markers for depression and bipolar disorder^1, 2^. But, in this study, we found that these four identified biomarkers had no sex specificity. As shown in the figure 2, the four identified markers could effectively distinguish female patients from female HCs with AUC of 0.993 (95% confidence interval (CI): 0.977-1), and male patients from male HCs with AUC of 0.961 (95% CI: 0.914-1).


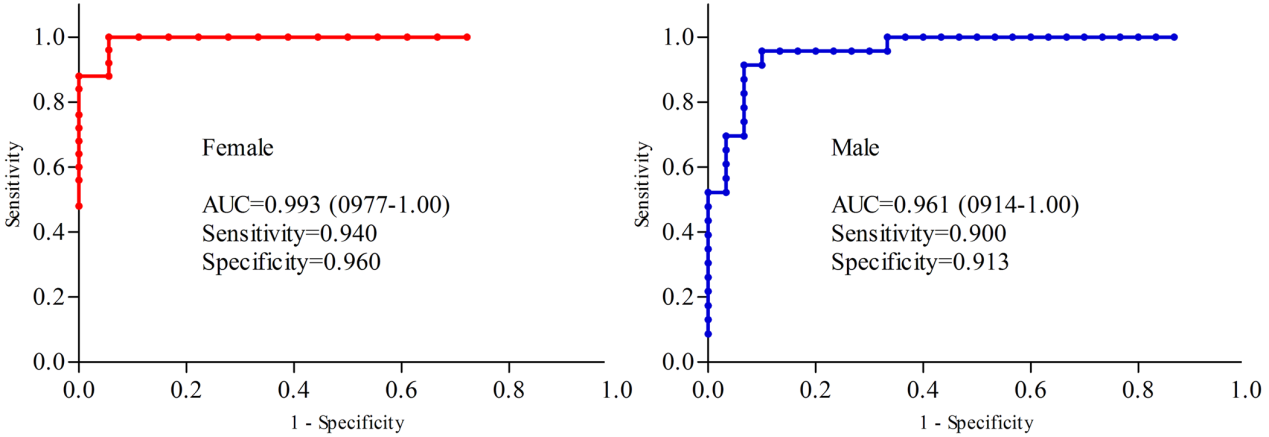


**Figure 2 Diagnostic performances of the panel in diagnosing female and male patients**

**Medication effects**

To determinate the homogeneity of metabolic phenotypes between the medicated and non-medicated patients, we firstly built the OPLS-DA model using the non-medicated patients and HCs (Figure 3A). Then, the constructed OPLS-DA model was used to predict class membership of the medicated patients (Figure 3B). As shown in Figure 3, the medicated and non-medicated patients had similar metabolic phenotypes, which suggested that medication might have little impact on metabolites in urine.

**
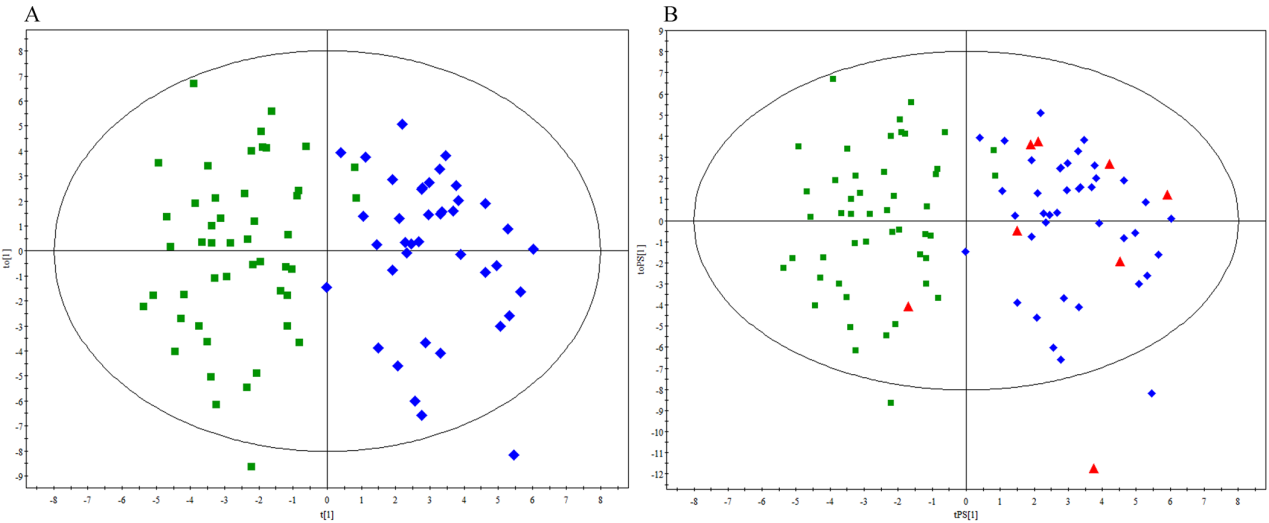
**

**Figure 3 Metabolic phenotypes homogeneity in non-medicated and medicated patients**

**NMR Acquisition**

Prior to NMR analysis, urine samples were thawed and centrifuged at 1500 g for 10 min to remove precipitation. To ensure stabilization of urinary pH, 500 µl of urine was mixed with 100 µl of phosphate buffer (90% D_2_O, 1 mM 3-trimethylsilyl-1-[2, 2, 3, 3-²H4] propionate (TSP), and 3 mM sodium azide; pH 7.4). After centrifugation at 12000 rpm for 10 min, 500 µl samples of supernatant were transferred into 5 mm NMR tubes. The proton spectra of the urine samples were collected on a Bruker Avance 600 spectrometer operating at a 600.13 MHz ^1^H frequency with a standard 1-dimensional (1D) pulse sequence. Typically, 64 transients were collected into 16K data points with a spectral width of 8000 Hz, an acquisition time of 0.945 s, and a relaxation delay of 2 s. Prior to Fourier transformation, the free induction decay (FID) was zero-filled and multiplied by an exponential function corresponding to a line-broadening factor of 0.3 Hz in the frequency domain. Urine resonance assignments were performed according to previous literature and NMR databases^3, 4^.

**GC-MS Acquisition**

The procedure for GC-MS preparation was performed according to our previous study^5^. Briefly, a 15 µl aliquot of urine was vortexed after adding 10 µl internal standard solutions (L-leucine-13C6, 0.02 mg/ml). Then, 15 µl urease was added into this mixed solution. The urea was degraded for 60 min at 37°C. The mixture was extracted with 240 μl of ice-cold methanol and then 80 μl of ice-cold methanol. After vortexing for 30 s, the mixture was centrifuged at 14000 rpm for 5 min at 4°C. The 224 ul supernatant was transferred to a glass vial and vacuum-dried at room temperature. The dried metabolic extract was derivatized with 30 µl of methoxyamine (20 mg/ml) for 1.5 h at 37°C. Subsequently, 30 µl of BSTFA with 1% TCMS was added into the mixture and heated for 1 h at 70°C, forming trimethylsilyl (TMS) derivatives. After derivatization and cooling to room temperature, 1.0 µl of this derivative was injected into the GC/MS for analysis. GC/MS analysis was carried out according to this group’s previously published work^6^.

**References**

1. Chen JJ, Huang H, Zhao LB, Zhou DZ, Yang YT, Zheng P, et al. Sex-Specific Urinary Biomarkers for Diagnosing Bipolar Disorder. *Plos One* 2014; **9**(12):e115221.

2. Zheng P, Chen JJ, Zhou CJ, Zeng L, Li KW, Sun L, et al. Identification of sex-specific urinary biomarkers for major depressive disorder by combined application of NMR- and GC–MS-based metabonomics. *Translational Psychiatry* 2016; **6**(11):e955.

3. Yap IK, Angley M, Veselkov KA, Holmes E, Lindon JC, Nicholson JK. Urinary metabolic phenotyping differentiates children with autism from their unaffected siblings and age-matched controls. J*ournal of proteome research* 2010; **9**(6): 2996-3004.

4. Zheng P, Wang Y, Chen L, Yang D, Meng H, Zhou D*, et al*. Identification and validation of urinary metabolite biomarkers for major depressive disorder. *Molecular & cellular proteomics: MCP* 2013; **12**(1)**:** 207-214.

5. Zheng P, Chen JJ, Huang T, Wang MJ, Wang Y, Dong MX, et al. A novel urinary metabolite signature for diagnosing major depressive disorder. *Journal of proteome research* 2013; **12**(12): 5904-5911.

6. Wei-Hua Shao, Song-Hua Fan, Yang Lei, Guo-En Yao, Jian-Jun Chen, Jian Zhou, et al. Metabolomic identification of molecular changes associated with stress resilience in the chronic mild stress rat model of depression. *Metabolomics* 2012; **9**(2): 433-443.
